# *Tubulinosema* sp. Microsporidian Myositis in Immunosuppressed Patient

**DOI:** 10.3201/eid1709.101926

**Published:** 2011-09

**Authors:** Maria M. Choudhary, Maureen G. Metcalfe, Kathryn Arrambide, Caryn Bern, Govinda S. Visvesvara, Norman J. Pieniazek, Rebecca D. Bandea, Marlene DeLeon-Carnes, Patricia Adem, Moaz M. Choudhary, Sherif R. Zaki, Musab U. Saeed

**Affiliations:** Author affiliations: Cleveland Clinic Foundation, Cleveland, Ohio, USA (Maria M. Choudhary);; Centers for Disease Control and Prevention, Atlanta, Georgia, USA (M.G. Metcalfe, C. Bern, G.S. Visvesvara, N.J. Pieniazek, R.D. Bandea, M. DeLeon-Carnes, P. Adem, S.R. Zaki);; Southern Illinois University, Quincy, Illinois, USA (K. Arrambide, Moaz M. Choudhary, M.U. Saeed)

**Keywords:** fungal infections, microsporidia, Tubulinosema, immunosuppression, myositis, dispatch

## Abstract

The Phylum Microsporidia comprises >1,200 species, only 15 of which are known to infect humans, including the genera *Trachipleistophora*, *Pleistophora*, and *Brachiola*. We report an infection by *Tubulinosema* sp. in an immunosuppressed patient.

Initially designated as primitive eukaryotic protozoa, the microsporidia are now classified as fungi (*1**,**2*) with >1,200 known species. The ribosomes of microsporidia resemble prokaryotic ribosomes in terms of size but lack a 5.8S subunit (*3*). The microsporidia infect many different animals and insects, but human infections were rarely reported before the HIV/AIDS epidemic when *Enterocytozoon bieneusi* was shown to be a major cause of diarrhea in patients with low CD4+ lymphocyte counts (*4*). Since then, 14 other species of microsporidia have been reported to infect the human gastrointestinal tract, eye, or muscle and to cause disseminated infection, most commonly in immunocompromised hosts (*5**–**12*). We report an infection by *Tubulinosema* sp. in an immunosuppressed patient.

## The Study

Our patient was a 67-year-old woman with known high-grade non-Hodgkin lymphoma since 1993 and chronic lymphocytic leukemia since 2003. She had received multiple courses of chemotherapy, including fludarabine, cyclophosphamide, rituximab, pentostatin, rituximab, cyclophosphamide, doxorubicin, vincristine, prednisolone, and alemtuzumab. During 2004 through 2008, she had multiple hospitalizations with neutropenic complications. Her chemotherapy regimen was switched to bendamustine and cyclophosphamide in January 2009 because of persistent neutropenia.

In February 2009, the patient was hospitalized with fever and abdominal pain. No clear infectious etiology could be ascertained despite an extensive workup; she was treated with cefepime, vancomycin, and metronidazole without any improvement. At the end of the antimicrobial drug course, she noticed 2 painful white lesions on her tongue and also had fever and chills. The lesions consisted of two 1.0- × 1.5-cm nodules on the anterior aspect of the tongue. These were initially treated as oral thrush with fluconazole without resolution. At the same time, the patient experienced a relapse of herpes zoster that was treated with valacyclovir.

In April 2009, biopsy samples of the lesions were obtained. Results of the initial histopathology report were consistent with an inflamed granulation type tissue with collections of epithelioid histiocytes resembling naked-type granulomatous changes. Numerous intracellular microorganisms were seen in the myocytes. Culture for bacteria and fungi was negative and culture for microsporidia was not attempted. Serologic test results for *Toxoplasma gondii* and *Trypanosoma cruzi* were also negative.

Paraffin-fixed tissue and slides were sent to the laboratories of the Parasitic Diseases Branch and the Infectious Disease Pathology Branch, Centers for Disease Control and Prevention (CDC), Atlanta, GA, USA. Detailed results of the evaluations performed at CDC are given below. On the basis of a preliminary diagnosis of microsporidia, the patient was treated with albendazole 400 mg daily without any improvement.

In June 2009, the patient sought treatment for decreased urine output and acute kidney injury attributed to acute interstitial nephritis of unknown etiology based on eosinophils in her urine. A renal biopsy showed lymphocytic infiltrates negative for CD5 and CD20 and positive for paired box gene-5 and p53 expression, consistent with Richter’s transformation of the kidney. She was also found to have anterior mediastinal lymphadenopathy, interval increase in splenomegaly, ascites, pleural effusions, and bilateral interstitial infiltrates. Specimens from a bronchoscopy did not show any evidence of malignancy. Cultures were negative for bacteria and fungi. The patient died the next day. The family chose not to have an autopsy done. The cause of death was transformed chronic lymphocytic leukemia with acute renal failure as a contributory cause.

**Figure 1:**
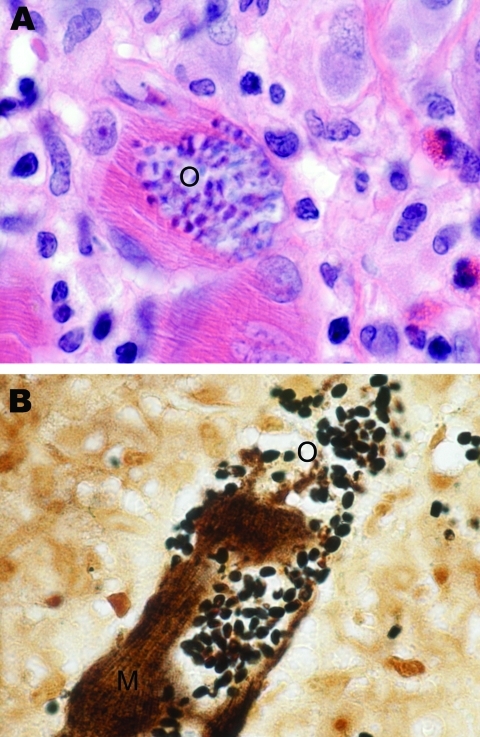
Skeletal muscle tissue samples from a 67-year-old woman with *Tubulinosema* sp. infection, 2009. A) Hematoxylin and eosin stain shows inflamed fibers with mononuclear infiltrate (O). B) Warthin-Starry silver stain shows abundant clusters of ovoid, basophilic organisms (O) within the muscle fibers (M). Original magnifications ×1,000.

Muscle tissue after fixation was stained with hematoxylin and eosin, periodic acid–Schiff, mucicarmine, Grocott methenamine silver, Giemsa, Warthin-Starry silver, acid-fast, and Lillie-Twort Gram stains. Granulomatous inflammation with focal infiltrates by neutrophils and eosinophils was seen. Within the myofibrils, there were abundant clusters of small, ovoid, basophilic organisms (Figure 1, panel A) measuring 2 µm stained positive by Lillie-Twort Gram and Warthin-Starry stains (Figure 1, panel B). The organisms stained faintly with Giemsa and were negative by Grocott methenamine silver, periodic acid–Schiff, and mucicarmine stains.

Results of immunohistochemical analysis (immune alkaline phosphatase technique) were negative, and autoimmune histochemical analysis was not performed. The primary antibodies used in the tests were an antibody against *T. cruzi* and an antibody against *T. gondii*. Appropriate positive and negative controls were run in parallel.

**Figure 2:**
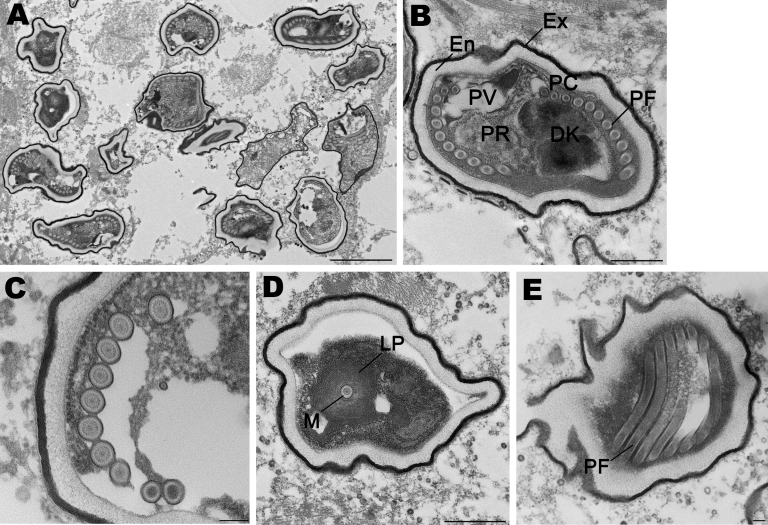
Spores of *Tubulinosema* sp. from a 67-year-old woman with *Tubulinosema* sp. infection, 2009. A) Electron micrograph of numerous spores in various stages in muscle tissue. Scale bar = 2 μm. B) An immature spore showing an electron-dense exospore (Ex) and a thick electron-lucent endospore (En), which together compose the spore wall. Diplokaryon (DK), posterior vacuole (PV), ribosomes in crystalline clusters as polyribosomes (PR) along with the polaroplast and polar filaments (PF) constitute the sporoplasm. A small difference in polar filament diameter in the anterior and posterior coils (PC), with the last 3 or 4 coils being smaller (anisofilar), is also apparent. Scale bar = 500 nm. C) Polar filaments exhibiting a lucent ring around a dense core. Scale bar = 100 nm. D) Cross-section of the manubroid (M), which is the straight portion of the polar filament surrounded by the lamellar polaroplast (LP). Scale bar = 500 nm. E) Longitudinal sections of polar filaments (PF) stained with uranyl acetate-lead citrate. Scale bar = 100 nm.

Electron microscopy at CDC revealed numerous spores but few developing stages (Figure 2, panel A). All stages were in direct contact with the host cell cytoplasm. The spores ranged from 1.4 to 2.4 μm in length and were characterized by an outer electron-dense exospore and a thick electron-lucent endospore. Within the endospore, a thin plasma membrane surrounded the polar filament coils and a polaroplast. These features are diagnostic characteristics of microsporidia. The endospore was considerably thinner near the anchoring disk. The polar filament had 11 coils arranged mostly in single rows, although in a few spores double rows were also seen. Three of the coils were slightly smaller than the others, which indicated the polar filaments are anisofilar (Figure 2, panel B). The polar filament coils measured 83.3–102 nm. At higher magnification, the polar filament coil exhibited a lucent ring around a dense core (Figure 2, panel C). A salient feature of the spore was the presence of a diplokaryotic nucleus with 2 nuclei closely opposed in a coffee bean–like appearance. The cytoplasm surrounding the nucleus was densely packed with ribosomes. Additional morphologic features included a posterior vacuole (Figure 2, panel B) and lamellar polaroplast consisting of tightly coiled membranes encircling the polar filament (Figure 2, panel D).

Molecular analysis was performed on a specimen of human muscle only. Taxonomically, the isolated spores’ small subunit of rRNA sequence on BLAST analysis (www.ncbi.nlm.nih.gov/BLAST) was found to be 100% identical (within a 500-nt sequenced fragment) to *Tubulinosema acridophagus* (GenBank accession no. AF024658), which usually infects North American grasshoppers (*Schistocerca americana* and *Melanoplus* spp.). It was also 96% identical to that of *Tu. ratisbonensis* (GenBank accession no. AY695845) obtained from a *Drosophila melanogaster* fruit fly and to *Tu. kingi* (GenBank accession no. DQ019419 and L28966) obtained from a *D. willistoni* fruit fly (Table).

**Table:** Comparison of various *Tubulinosema* spp. with organism isolated from a 67-year old woman in 2009*

Characteristics	*Tu. ratisbonensis*	*Tu. kingi*	*Tu. acridophagus*	2009 isolate
First described	2005	1962	1967	NA
Previously named	NA	*Nosema kingi*	*Visvesvaria acridophagus*, *Nosema acridophagus*	NA
Host in which identified	*Drosophila melanogaster* fruit fly (Diptera)	*D. willistoni* fruit fly	*Schistocerca Americana* grasshopper (Orthoptera)	Human (skeletal muscle)
Known human infections	None	None	None	None
Microtubules on plasma lemma	Present	Present	Present	Not seen
Spore shape	Pear shaped	Oval	Oval	Round-to-pear shaped
Meronts nuclei	1, 2, or 4	1, 2, or 4	1, 2, or 4	Meronts not seen
Sporonts nuclei	2–4	2–4	2–4	Sporonts not seen
Polar tube	9–14	13	10–12	11
Coils/rows	Single	Single/ double (anterior)	Single	Single
Polar filament arrangement	Anisofilar	Isofilar	Isofilar	Anisofilar

*NA, not applicable.

## Conclusions

We report a case of microsporidian myositis caused by *Tubulinosema* sp. in a patient with chronic lymphocytic leukemia and subsequent Richter’s transformation. Franzen et al. in 2005 proposed a new genus and species (*13*), *Tu. ratisbonensis*, for a microsporidium that parasitizes the fruit fly *D. melanogaster*. Subsequently, phylogenetic analyses of ribosomal RNA sequences determined that *Tu. ratisbonensis* was similar to several species included in the genus *Nosema* (*13*). Two other species of *Nosema* (*N. kingi* and *N. acridophagus*), both parasites of insects fitting the generic description of *Tubulinosema*, were transferred to the new genus (*13*)*.* On the basis of limited ultrastructural studies, the microsporidia described here resemble *Tu. acridophagus*, *Tu. kingi*, and *Tu. ratisbonensis* (Table). Moreover, PCR of formalin-fixed, paraffin-embedded tissue showed that it was closely related to *Tu. acridophagus*, a parasite of the fruit fly *D. melanogaster*, suggesting an insect source of this infection.

To the best of our knowledge, before this case microsporidia belonging to the genus *Tubulinosema* had not been associated with human infection. However, members of another genus *Anncaliia* (*Brachiola*), recently classified under the same family *Tubulinosematidae* as *Tubulinosema*, have been known to cause myositis and keratitis in humans (*14*). *A. algerae* is a well known parasite of mosquitoes and has also been described as causing infections in humans (*15*). Currently, microsporidia belonging to 8 genera are known to cause human infections (*4*). Therefore, clinicians managing immunodeficient patients who have fatigue, weakness, and other nonspecific symptoms, including unexplainable lesions, should consider microsporidiosis as a possible differential diagnosis.
